# S108. ASSOCIATIONS BETWEEN GLOBAL BRAIN MEASURES AND STATE- AND TRAIT- RELATED SYMPTOM EXPRESSION OF SCHIZOPHRENIA

**DOI:** 10.1093/schbul/sby018.895

**Published:** 2018-04-01

**Authors:** Laila Asmal, Stéfan Du Plessis, Bonginkosi Chiliza, Sanja Kilian, Riaan Olivier, Freda Scheffler, Lebogang Phahladira, Paola Dazzan, Robin Emsley

**Affiliations:** 1 Stellenbosch University; 2 Institute of Psychiatry, Psychology and Neuroscience, King’s College London

## Abstract

**Background:**

The course of schizophrenia is characterised by episodes of psychotic symptoms and enduring deficits of negative symptoms, cognition and functioning. We investigated the relationship between global brain measures and trait-related symptoms (endpoint scores), and global brain measures and state-related symptoms (change scores).

**Methods:**

We examined global cortical, subcortical and white matter volume, and global cortical thickness in 54 first-episode schizophrenia patients at baseline. We performed clinical, cognitive, and neurological assessments at baseline and twelve month follow-up. We used hierarchical multiple regression to predict baseline brain measures.

**Results:**

State-related clinical predictors accounted for 8% of variance in white matter volume, trait-related clinical predictors accounted for 7% of variance in subcortical volume. Trait-related cognitive scores accounted for 15% of variance in subcortical volume and 13% of variance in cortical volume. Baseline subcortical gray matter volume was significantly associated with sensory integration (0.02) and verbal learning (0.04) trait scores, cortical volume with verbal learning (0.04) trait scores, cortical thickness with social and occupational functioning (0.03) trait scores, and white matter volume with motor coordination (0.007) state scores.

**Discussion:**

Impaired verbal learning may be the cognitive domain that is particularly trait-related, and possibly closest to the neurodevelopmental deficit underlying schizophrenia. State and trait components of neurological soft signs may be differentially related to brain structure. Mediators of the relationship between trait functional deficits and cortical thickness needs consideration.

